# Supplementary material for: Mapping the evolution of fertility support policies in China: A content and instrumental analysis
Source: PLoS One. 2025 Oct 9;20(10):e0332137. doi: 10.1371/journal.pone.0332137 (PMC12510515; doi:10.1371/journal.pone.0332137)
Supplement: S1 Appendix — (ZIP) [file pone.0332137.s001.zip › S1 Appendix. 226 original policy documents/113-人力资源社会保障部、财政部、自然资源部等关于进一步用好公益性岗位发挥就业保障作用的通知(FBM-CLI-4-342809).doc]

人力资源社会保障部、财政部、自然资源部等关于进一步用好公益性岗位发挥就业保障作用的通知

**发布部门：** [人力资源和社会保障部](https://www.pkulaw.com/law/chl?Aggs.IssueDepartment=60236) [财政部](https://www.pkulaw.com/law/chl?Aggs.IssueDepartment=60210) [自然资源部](https://www.pkulaw.com/law/chl?Aggs.IssueDepartment=60243) [交通运输部](https://www.pkulaw.com/law/chl?Aggs.IssueDepartment=60235) [水利部](https://www.pkulaw.com/law/chl?Aggs.IssueDepartment=60218) [国家林业和草原局](https://www.pkulaw.com/law/chl?Aggs.IssueDepartment=60428) [国务院扶贫办](https://www.pkulaw.com/law/chl?Aggs.IssueDepartment=7060426) [机构沿革](javascript:void(0))

**发文字号：**人社部发〔2020〕38号

**发布日期：**2020.05.29

**实施日期：**2020.05.29

**时效性：** [现行有效](https://www.pkulaw.com/law/chl?Aggs.TimelinessDic=01)

**效力级别：** [部门规范性文件](https://www.pkulaw.com/law/chl?Aggs.EffectivenessDic=XE0302)

**法规类别：** [招工与就业](https://www.pkulaw.com/law/chl?Aggs.Category=08702) [扶贫救灾救济](https://www.pkulaw.com/law/chl?Aggs.Category=01504) [疫情防控](https://www.pkulaw.com/law/chl?Aggs.Category=122)

**专题分类：** [突发事件](https://www.pkulaw.com/law/chl?Aggs.SpecialType=005)

人力资源社会保障部、财政部、自然资源部、交通运输部、水利部、国家林业和草原局、国务院扶贫办关于进一步用好公益性岗位发挥就业保障作用的通知
（人社部发〔2020〕38号）

各省、自治区、直辖市及新疆生产建设兵团人力资源社会保障厅（局）、财政厅（局）、自然资源厅（局）、交通运输厅（局）、水利（水务）厅（局）、林业和草原主管部门、扶贫办（局）：
　　就业事关基本民生、经济发展和社会稳定大局。为落实党中央、国务院决战决胜脱贫攻坚、应对新冠肺炎疫情影响强化稳就业决策部署，进一步用好各类公益性岗位，充分发挥就业保障作用，现就有关事项通知如下：
　　**一、**切实提高思想认识。各地要高度重视公益性岗位有关工作，将其作为当前今后一段时期稳定和扩大就业的重要举措，摆在更加突出位置。压实工作责任，加强资金保障，推动政策落实，强化日常调度与集中督查。统筹各方资源，形成工作合力，加强部门间协同配合，切实发挥公益性岗位“兜底线、救急难”作用。

　　**二、**做好就业困难人员认定。根据本地区经济社会发展和就业形势变化，动态调整就业困难人员认定标准，及时将受疫情影响失业人员、残疾人员、建档立卡贫困劳动力等各类就业困难人员纳入援助对象范围。推动就业困难人员认定与失业登记协同办理，便捷受理申请，同步开展认定，主动提供援助服务，做到应认尽认、应帮尽帮。

　　**三、**多渠道开展就业援助。根据就业援助对象特点和需求，设计服务路径和援助举措，实施分类帮扶。针对性开展职业技能培训，积极推荐企业吸纳，帮助灵活就业，扶持自主创业，落实好税费减免、社会保险补贴、职业培训补贴等政策。对仍然难以通过市场渠道实现就业的，通过公益性岗位予以安置。

　　**四、**聚焦城乡公共服务短板把握开发领域。围绕疫情防控等重大突发事件，开发防疫消杀、医护辅助、物资配送、道路管制、卡点值守等应急管理服务岗位。满足城乡基层公共服务需求，开发保洁、保绿、公共设施维护、便民服务、妇幼保健、托老托幼助残、乡村快递收发等便民服务类岗位。弥补“三农”领域基础设施建设、人居环境整治和生态治理不足，开发农村公路建设与管护、村庄公共基础设施建设与管护、水利工程及水利设施建设与管护、河湖巡查与管护、垃圾污水处理、厕所粪污无害化处理、河塘清淤整治、造林绿化等岗位。加强与各类公共服务管理主管部门的沟通联系，掌握相关领域招人用人需求，拓展岗位开发范围。

　　**五、**合理利用临时性城镇公益性岗位。2020年新开发的城镇公益性岗位以短期性、临时性岗位为主，提升资金使用效率，合理安置人员。临时性公益性岗位在岗时间不超过6个月，探索实施项目制管理，适当采取非全日制等方式。根据实际工作时间等因素合理确定岗位补贴，全日制岗位补贴不超过当地月最低工资标准，非全日制岗位补贴不超过当地小时最低工资标准，社会保险补贴根据参加社会保险情况按规定发放。

　　**六、**协同协力用好乡村公益性岗位助力脱贫攻坚。加强乡村公益性岗位开发管理部门间的协同配合，定期开展信息共享，汇聚各类岗位合力，助力脱贫攻坚。充分考虑当地收入水平和岗位职责内容，合理确定岗位待遇水平。指导乡镇、村结合实际开展岗位招聘，将岗位更多用于安置无法外出、无业可扶、无力脱贫的建档立卡贫困劳动力。通过现有资金渠道，加大对现有各类乡村公益性岗位的支持力度，并结合实际实行动态调整。

　　**七、**公开公平公正开展岗位招聘。公开发布岗位招聘信息，在网络或街道（乡镇）、社区（村）公告栏发布张贴招聘公告，注明岗位职责、招聘要求、薪酬待遇、劳动时长、在岗时间等信息。规范开展人员招用，组织好上岗资格认定、人岗匹配和拟招用人员公示等工作，接受社会各方监督。优化服务方式，搭建信息发布平台，开设招聘服务专区，为公益性岗位用人单位和劳动者提供更加优质高效的求职招聘服务。

　　**八、**强化岗位规范化管理。严格开展安置人员身份核实认定，确保依法依规安置符合条件人员，强化相关补贴资金监管。加强在岗人员履职情况监管，定期开展考核评价，重点考核工作成效、遵守规章制度和工作纪律情况。及时纠正查处安置不符合条件人员、优亲厚友、轮流坐庄、资金补贴一发了之、变相发钱等违法违规行为，坚决避免一村多岗、一人多岗等岗位设置过多过滥等现象。强化实名制动态管理，建立省级公益性岗位数据库，做好各类公益性岗位统计分析，按季度报送有关情况。

　　**九、**加强组织领导。各地要围绕稳定和扩大就业工作目标，立足职责、密切合作。人力资源社会保障部门会同扶贫部门加强各类公益性岗位统计分析，摸清就业困难人员、建档立卡贫困劳动力等困难群体就业状况和就业需求，会同财政部门做好就业相关资金补助公益性岗位相关政策制定。扶贫部门加大光伏收益、扶贫专项资金开发乡村公益性岗位力度，会同人力资源社会保障部门、自然资源部门、交通运输部门、水利部门、林业草原部门做好各类乡村公益性岗位的统筹利用。交通运输部门做好护路员岗位的开发管理，协同做好岗位招聘与安置人员统计分析。水利部门做好水利工程建设与管护岗位管理，协同做好岗位招聘和安置人员统计分析。各地财政部门会同人力资源社会保障部门、自然资源部门、交通运输部门、水利部门、林业草原部门、扶贫部门做好各类公益性岗位资金支出使用情况的监管检查。

人力资源社会保障部
财政部
自然资源部
交通运输部
水利部
国家林业草原局
国务院扶贫办
2020年5月29日

© 北大法宝：（[www.pkulaw.com](https://www.pkulaw.com/)) 专业提供法律信息、法学知识和法律软件领域各类解决方案。北大法宝为您提供丰富的参考资料，正式引用法规条文时请与标准文本核对。
欢迎查看所有[产品和服务](https://www.pkulaw.com/helps/2.html)。法宝快讯：[如何快速找到您需要的检索结果？](https://www.pkulaw.com/helps/70.html)[法宝](https://www.pkulaw.com/helps/69.html)V6有何新特色？

扫描二维码阅读原文

**原文链接：**<https://www.pkulaw.com/chl/e9ef5c9d019c2248bdfb.html>
